# Lateral fixation: an alternative surgical approach in the prevention of complete atypical femoral fractures

**DOI:** 10.1007/s00590-017-2041-6

**Published:** 2017-09-18

**Authors:** Mohammad Kharazmi, Karl Michaëlsson, Pär Hallberg, Jörg Schilcher

**Affiliations:** 1 Section of Orthopaedics, Department of Surgical Sciences, Uppsala University, 751 85 Uppsala, Sweden; 2 Department of Medical Sciences, Uppsala University, Uppsala, Sweden; 3 Section of Orthopaedics, Department of Clinical and Experimental Medicine, Faculty of Health Sciences, Linköping University, Linköping, Sweden; 4 Department of Oral and Maxillofacial Surgery, Västmanland Hospital, Västerås, Sweden

**Keywords:** Atypical fracture, Femoral fracture, Bisphosphonate, Osteoporosis, Fracture prevention

## Abstract

Little evidence is available on how to treat incomplete atypical fractures of the femur. When surgery is chosen, intramedullary nailing is the most common invasive technique. However, this approach is adopted from the treatment of other types of ordinary femoral fracture and does not aim to prevent the impending complete fracture by interrupting the mechanism underlying the pathology. We suggest a different surgical approach that intends to counteract the underlying biomechanical conditions leading to a complete atypical fracture and thus could be better suited in selected cases. Here, we share an alternative surgical approach and present two cases treated accordingly.

## Introduction

**Fig. 1:**
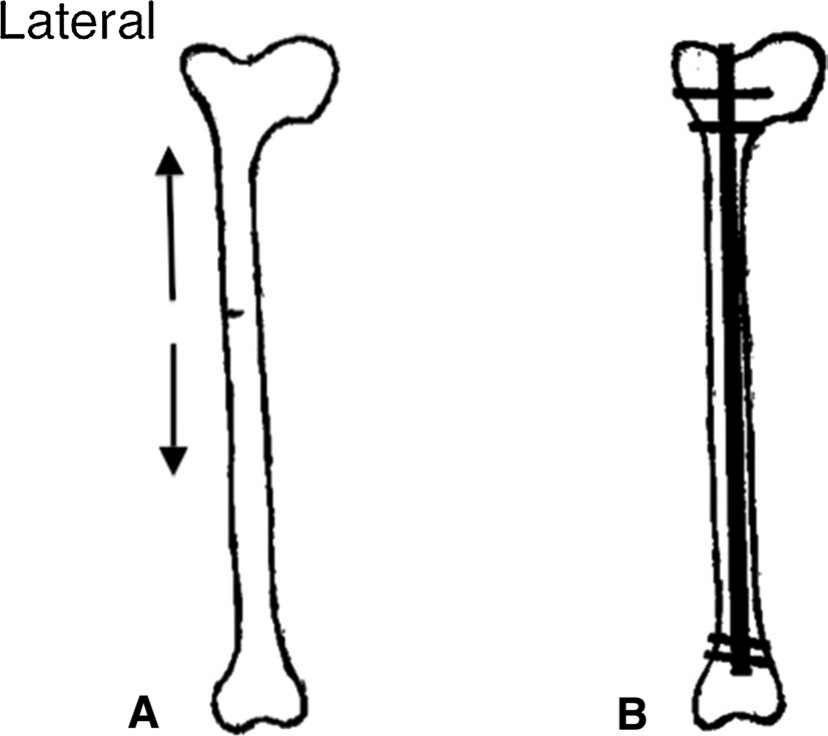
**a** Schematic drawing of an incomplete fracture confined to the lateral side of a femur with a centered axis (no curvature). Tensile forces applied to the lateral cortex are outlined (arrows). **b** Schematic drawing of the same femur as in **a** that was provided prophylactic treatment with an intramedullary nail (IMN) to prevent future completion of the atypical fracture

On plain radiographs, incomplete atypical femoral fractures (AFFs) can be seen as a horizontal radiolucent line confined to the lateral cortex of the affected femur (Fig. 1a). Further progression of the pathology leads to an extension of the crack medially, perpendicular to tensional forces in the femur. Ultimately, a complete fracture occurs involving the medial cortex with a typical spike [1]. The incomplete fracture is likely the most solid evidence of a pathological process already initiated. It is among the very few warning signs that may be presented to a healthcare provider and offers a window of opportunity in which a complete atypical fracture can still be prevented.

Despite previous concerns of delayed healing, several studies have reported positive outcomes for surgical treatment of complete AFFs [2, 3]. In contrast, there is little doubt that the ability to heal is somehow compromised in incomplete fractures [4, 5]. Healing rates for incomplete fractures treated without surgical fixation are low [6–8], and there are cases that have lasted for years without healing, despite cessation of bisphosphonate treatment [9].

**Fig. 2:**
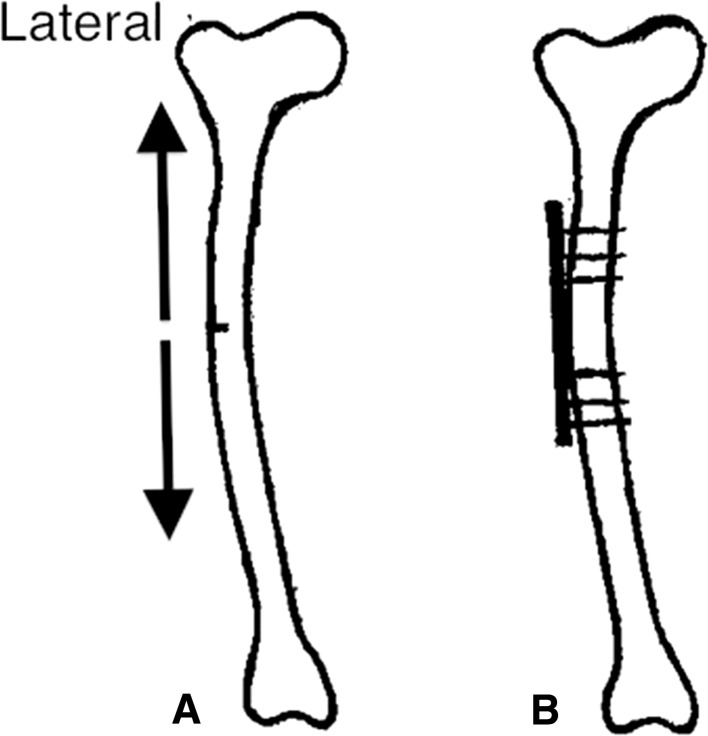
**a** Schematic drawing of a curved femur with incomplete fracture confined to the lateral side of the bone. Note: increased tensile forces (arrows) are applied to this femur compared with one with a centered axis (Fig. 1a). Because of the prominent curvature of this femur, it would be difficult to insert an intramedullary nail (IMN) (**b**) without the risk of causing further injury to the architecture of the bone. **b** The curved femur with an incomplete fracture that was provided prophylactic treatment with lateral fixation according to the present approach. The plate is positioned with six bicortical screws

Surgical fixation is successful in the treatment of incomplete AFFs [6, 10–12], and in this context intramedullary nails (IMNs) (Fig. 1b) are widely considered [6, 10] the surgical treatment of choice. However, the choice of IMNs is largely based on its empirical merits rather than being a surgical technique addressing the mechanism of AFFs. Here, we present an alternative surgical approach according to biomechanical considerations. The approach is tailored to counteract the mechanical forces that might result in the formation of a complete fracture and may be considered for patients with severe femoral curvature (Fig. 2a) or in patients with preexisting joint implants of the hip or knee.

In humans, the mean radius of curvature of the femur is 112 cm (SD = 26) [13]. However, the degree of individual variation is large and strongly influenced by ethnicity [13, 14]. Differences in the presence of a significant curvature relative to ethnicity have also been observed in patients with AFFs, ranging from 25% for females in Sweden to 45% for females in Singapore [3]. The current designs of IMNs are straighter than the average human femur, leading to a higher risk of cortical impingement with increasing curvature [15–18]. The mismatch may also lead to iatrogenic fractures during insertion of the nail, malalignment of the bone and delayed union [19, 20]. Such iatrogenic fractures appear to occur quite frequently when using traditional nails [21], especially in patients with incomplete AFF in which the femoral structure is intact. For these patients, the risk of iatrogenic fractures will be high. This problem with traditional nails is troublesome in view of the increasing number of studies revealing an association between femoral curvature and the risk of an AFF [22, 23].

In patients with preexisting joint replacement, particularly those with femoral stems in total hip arthroplasty and stemmed femoral components in total knee replacement, atypical fractures tend to occur in areas of stress concentrations at the tip of the implant [24, 25]. Moreover, the likelihood of crack propagation is high because of these stress concentrations. Because the intramedullary canal is occupied by the prosthetic stem, intramedullary fixation is impossible. Therefore, in this selected group of patients, we see the need for a preventive surgical intervention with a low risk of complications.

## Lateral fixation of the incomplete atypical fracture

**Fig. 3:**
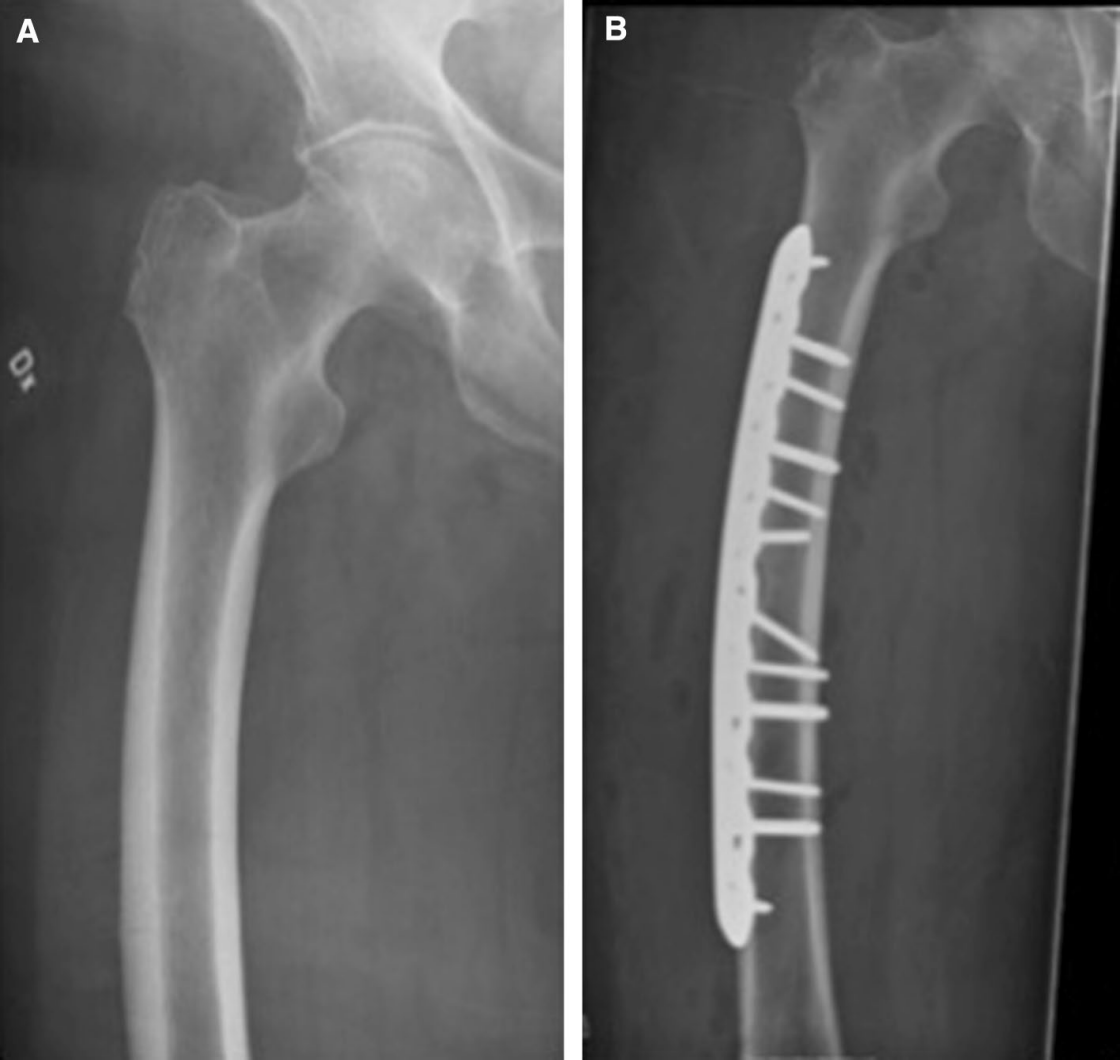
**a** An 80-year-old female sustained an incomplete fracture of her right femur. Before the fracture, she had received 5 years of treatment with alendronic acid because of a high dose of corticosteroids for rheumatic disease. She recalled enduring 6 months of increasing pain from her right thigh before seeking medical advice. **b** Surgery was selected as the preventive treatment of choice. Bisphosphonate treatment was discontinued before surgery. Lateral fixation was performed because of the curvature of the femur (femoral angle approximately 10°). Full weight bearing was allowed postoperatively. Radiographic examination after surgery revealed no further propagation of the fracture

**Fig. 4:**
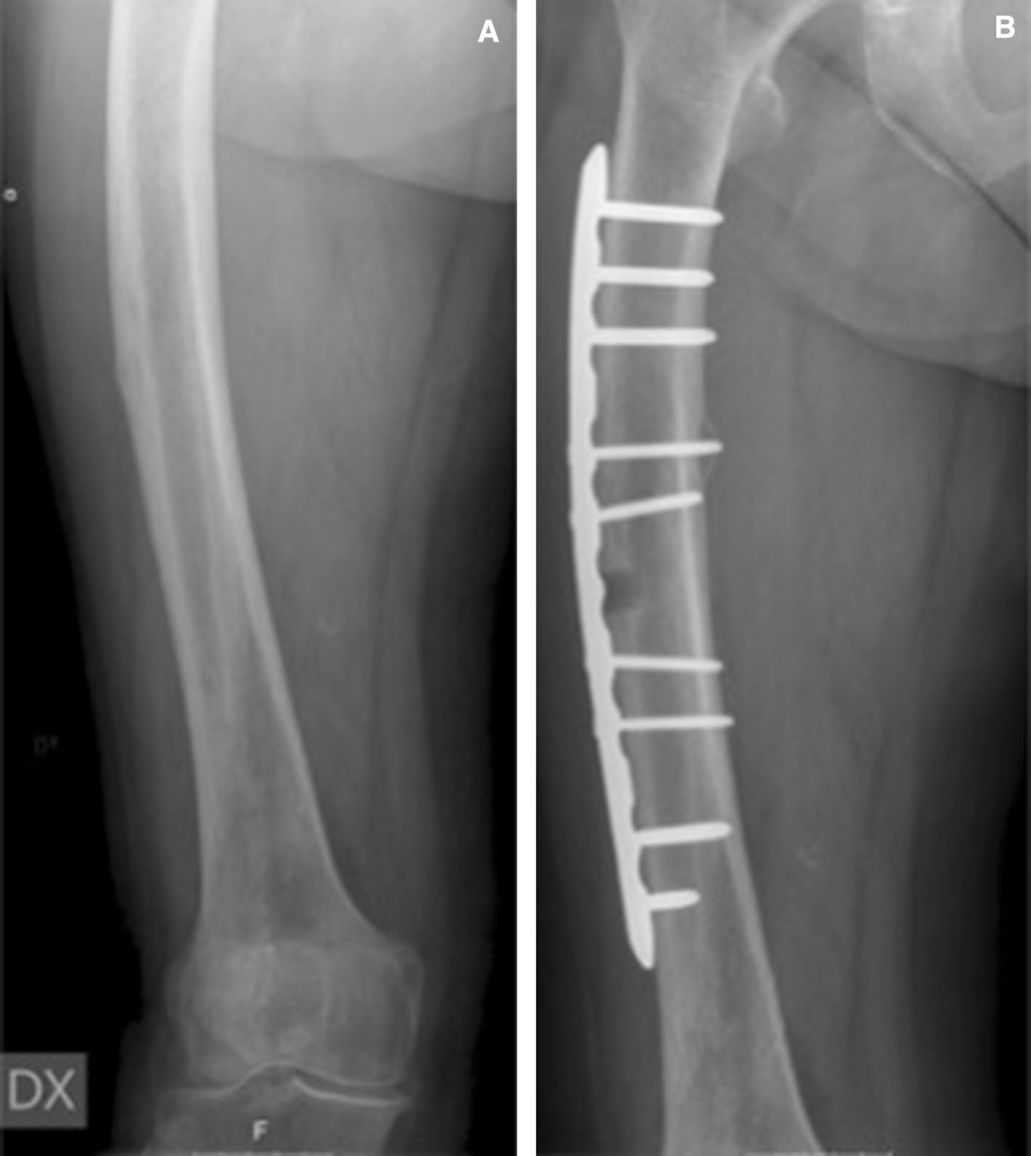
**a** An 83-year-old female sustained an incomplete fracture of her right femur without any history of previous bisphosphonate use. She recalled having 12 months of increasing pain from her right thigh before seeking medical attention. **b** Surgery was selected as the preventive treatment. Because of the curvature of the femur (femoral angle approximately 10°), lateral fixation was performed. A biopsy of the fracture site was taken to exclude other related conditions that might have contributed to the development of a stress fracture despite femoral bow. The defect created by the biopsy showed callus formation after 3 months and complete recortication after approximately 18 months. Full weight bearing was allowed postoperatively

The proposed surgical intervention is based on compression and fixation of the incomplete fracture in the lateral cortex with a plate (Fig. 2b). With vertical load, such as walking, the curved femur creates a tensile force laterally and a compressive force medially [26–28]. With the plate positioned laterally, its effect is similar to a tension band preventing further widening of the crack and reducing the risk of crack propagation (Fig. 2b). We have successfully applied this approach in two patients with curved femurs (Figs. 3, 4; Table 1).

**Table 1:** Summary of available data on lateral fixation of incomplete atypical femoral fractures (four patients, five femurs)

	Age/sex	Duration of bisphosphonate use (per os)	Prodromal symptoms	Femoral curvature	Surgical treatment	Functional recovery postsurgery
Tsuchie et al., case 1	78F	4 years	Ipsilateral thigh pain for 1 month	12° (lateral) 11° (anterior)	Lateral fixation with locking plate and six bicortical screws	Able to walk without pain after 2 weeks
Tsuchie et al., case 2	77F	6 years	Bilateral thigh pain for 6 months	Right femur:	Right femur: Lateral fixation with locking plate and six bicortical screws	Able to walk without pain after 3 weeks
				17° (lateral)		
				15° (anterior)		
				Left femur:	Left femur: Lateral fixation with locking plate and six bicortical screws	
				12° (lateral)		
				15° (anterior)		
Present article, case 1	80F	5 years	Ipsilateral thigh pain for 6 months	10° (lateral)	Lateral fixation with locking plate and 10 bicortical screws	Full weight bearing postoperatively
Present article, case 2	83F	No previous bisphosphonate use	Ipsilateral thigh pain for 12 months	10° (lateral)	Lateral fixation with locking plate and eight bicortical screws	Full weight bearing postoperatively

## Discussion

Several authors have reported on successful conservative approaches for incomplete AFFs [29, 30]. However, recognizing that a large proportion of incomplete AFFs will progress to complete fractures without surgical fixation mandates prophylactic surgical intervention [7, 8]. Prophylactic treatment offers several benefits, including shorter operation time, reduced bleeding and shorter postoperative hospitalization [7, 31].

Lateral fixation is already a documented approach for complete AFFs, where IMNs have proven to be less effective [25]. Its use in the prevention of complete AFFs is seldom reported. The current literature describes successful treatment in two patients with incomplete AFFs and significant curvature of the femurs (Table 1) [32]. Our results add further to this finding by showing that the technique is reproducible in the hands of other surgeons.

There are two main goals for the present approach. The first is to avoid a complete AFF. So far, we lack an understanding of the mechanism(s) underlying AFFs. However, accumulating evidence supports the notion that long-term bisphosphonate treatment may deteriorate the mechanical properties of the cortical bone that would lead to the formation of micro-cracks in the lateral cortex [33]. Such changes could be caused by a reduction in the mineral and matrix heterogeneity of the cortical bone, causing deterioration of tissue-level toughening mechanisms and inhibition of the mechanism of targeted remodeling [34].

Classical and more recent biomechanical analyses show that the lateral cortex of the femur is exposed to high tensile stress during each step. This stress is dependent on the activity performed and the musculoskeletal architecture of the individual [35–37]. Tensional forces at the lateral side of the bone will strive to open any existing defects (cracks) in the cortex (Fig. 1a). These forces may favor the development of AFFs when the skeleton is exposed to bisphosphonates [23]. The importance of tensile forces is supported by the observation that atypical lesions of the femur are clustered at the region of maximal tensile loading and not at locations subjected to compressive loading [35]. Tensile forces are likely to have a greater impact in the curved femur (Fig. 2a), bringing about an increased risk of AFF [22, 23]. Lateral plate fixation might inhibit the formation of micro-cracks and the progression of an incomplete fracture.

The second goal is to enhance the possibility of healing incomplete fractures. Reduced healing capacity of incomplete AFFs can partly be explained by biomechanical factors in which daily low-impact activities are enough to cause strains that prohibit bone formation [38]. Accordingly, we believe that the healing process may benefit if this strain were significantly reduced. Both of our patients were allowed full weight bearing postoperatively and quickly recovered full walking abilities (Figs. 3b, 4b), suggesting successful healing, as reported in previous reports [32] (Table 1).

The lateral fixation can be successfully used as a surgical preventive measure for the curved femur affected by an incomplete AFF. Further investigations are desirable before the technique can be usually applied to incomplete AFFs beyond the curved femur.

## References

[1] Schilcher J, Koeppen V, Ranstam J, Skripitz R, Michaelsson K, Aspenberg P (2013). Atypical femoral fractures are a separate entity, characterized by highly specific radiographic features. A comparison of 59 cases and 218 controls. Bone.

[2] Egol KA, Park JH, Rosenberg ZS, Peck V, Tejwani NC (2014). Healing delayed but generally reliable after bisphosphonate-associated complete femur fractures treated with IM nails. Clin Orthop Relat Res.

[3] Schilcher J (2015). High revision rate but good healing capacity of atypical femoral fractures. A comparison with common shaft fractures. Injury.

[4] Harvey EJ (2016). Bisphosphonates are not always helpful: commentary on an article by Hae-Seong Lim, MD, et al.: “factors associated with increased healing time in complete femoral fractures after long-term bisphosphonate therapy”. J Bone Joint Surg Am.

[5] Ha YC, Cho MR, Park KH, Kim SY, Koo KH (2010). Is surgery necessary for femoral insufficiency fractures after long-term bisphosphonate therapy?. Clin Orthop Relat Res.

[6] Egol KA, Park JH, Prensky C, Rosenberg ZS, Peck V, Tejwani NC (2013). Surgical treatment improves clinical and functional outcomes for patients who sustain incomplete bisphosphonate-related femur fractures. J Orthop Trauma.

[7] Banffy MB, Vrahas MS, Ready JE, Abraham JA (2011). Nonoperative versus prophylactic treatment of bisphosphonate-associated femoral stress fractures. Clin Orthop Relat Res.

[8] Lee YK, Ha YC, Kang BJ, Chang JS, Koo KH (2013). Predicting need for fixation of atypical femoral fracture. J Clin Endocrinol Metab.

[9] Schilcher J, Sandberg O, Isaksson H, Aspenberg P (2014). Histology of 8 atypical femoral fractures: remodeling but no healing. Acta Orthop.

[10] Oh CW, Oh JK, Park KC, Kim JW, Yoon YC (2013). Prophylactic nailing of incomplete atypical femoral fractures. Sci World J.

[11] Markolf KL, Cheung E, Joshi NB, Boguszewski DV, Petrigliano FA, McAllister DR (2016). Plate versus intramedullary nail fixation of anterior tibial stress fractures: a biomechanical study. Am J Sports Med.

[12] Ward WG, Carter CJ, Wilson SC, Emory CL (2012). Femoral stress fractures associated with long-term bisphosphonate treatment. Clin Orthop Relat Res.

[13] Maratt J, Schilling PL, Holcombe S, Dougherty R, Murphy R, Wang SC, Goulet JA (2014). Variation in the femoral bow: a novel high-throughput analysis of 3922 femurs on cross-sectional imaging. J Orthop Trauma.

[14] Chapman T, Sholukha V, Semal P, Louryan S, Rooze M, Jan SVS (2015). Femoral curvature variability in modern humans using three-dimensional quadric surface fitting. Surg Radiol Anat.

[15] Buford WL, Turnbow BJ, Gugala Z, Lindsey RW (2014). Three-dimensional computed tomography-based modeling of sagittal cadaveric femoral bowing and implications for intramedullary nailing. J Orthop Trauma.

[16] Egol KA, Chang EY, Cvitkovic J, Kummer FJ, Koval KJ (2004). Mismatch of current intramedullary nails with the anterior bow of the femur. J Orthop Trauma.

[17] Scolaro JA, Endress C, Mehta S (2013). Prevention of cortical breach during placement of an antegrade intramedullary femoral nail. Orthopedics.

[18] Roberts JW, Libet LA, Wolinsky PR (2012). Who is in danger? Impingement and penetration of the anterior cortex of the distal femur during intramedullary nailing of proximal femur fractures: preoperatively measurable risk factors. J Trauma Acute Care Surg.

[19] Zbeda RM, Sculco PK, Urch EY (2015). Tension band plating for chronic anterior tibial stress fractures in high-performance athletes. Am J Sports Med.

[20] Yang KH, Min BW, Ha YC (2015). A typical femoral fracture: 2015 position statement of the Korean society for bone and mineral research. J Bone Metab.

[21] Park YC, Song HK, Zheng XL, Yang KH (2017). Intramedullary nailing for atypical femoral fracture with excessive anterolateral bowing. J Bone Joint Surg Am.

[22] Taormina DP, Marcano AI, Karia R, Egol KA, Tejwani NC (2014). Symptomatic atypical femoral fractures are related to underlying hip geometry. Bone.

[23] Morin SN, Wall M, Belzile EL (2016). Assessment of femur geometrical parameters using EOS imaging technology in patients with atypical femur fractures; preliminary results. Bone.

[24] Lee JY, Soh T, Howe TS, Koh JS, Kwek EB, Chua DT (2015). Bisphosphonate-associated peri-implant fractures: a new clinical entity?. Acta Orthop.

[25] Niikura T, Lee SY, Sakai Y, Kuroda R, Kurosaka M (2015). Rare non-traumatic periprosthetic femoral fracture with features of an atypical femoral fracture: a case report. J Med Case Rep.

[26] Polgár K, Gill HS, Viceconti M, Murray DW, O’Connor JJ (2003). Strain distribution within the human femur due to physiological and simplified loading: finite element analysis using the muscle standardized femur model. Proc Inst Mech Eng Part H J Eng Med.

[27] van der Meulen MC, Boskey AL (2012). Atypical subtrochanteric femoral shaft fractures: role for mechanics and bone quality. Arthritis Res Ther.

[28] Koeppen VA, Schilcher J, Aspenberg P (2013). Dichotomous location of 160 atypical femoral fractures. Acta Orthop.

[29] Saleh A, Hegde VV, Potty AG, Schneider R, Cornell CN, Lane JM (2012). Management strategy for symptomatic bisphosphonate-associated incomplete atypical femoral fractures. HSS J.

[30] Kim HS, Jung HY, Kim MO, Joa KL, Kim YJ, Kwon SY, Kim CH (2015). Successful conservative treatment: multiple atypical fractures in osteoporotic patients after bisphosphate medication: a unique case report. Medicine (Baltimore).

[31] Shaikh W, Morris D, Morris S (2016). Signs of insufficiency fractures overlooked in a patient receiving chronic bisphosphonate therapy. J Am Board Fam Med.

[32] Tsuchie H, Miyakoshi N, Nishi T, Abe H, Segawa T, Shimada Y (2015). Combined effect of a locking plate and teriparatide for incomplete atypical femoral fracture: two case reports of curved femurs. Case Rep Orthop.

[33] Bajaj D, Geissler JR, Allen MR, Burr DB, Fritton JC (2014). The resistance of cortical bone tissue to failure under cyclic loading is reduced with alendronate. Bone.

[34] Donnelly E, Meredith DS, Nguyen JT (2012). Reduced cortical bone compositional heterogeneity with bisphosphonate treatment in postmenopausal women with intertrochanteric and subtrochanteric fractures. J Bone Miner Res.

[35] Koh JS, Goh SK, Png MA, Ng AC, Howe TS (2011). Distribution of atypical fractures and cortical stress lesions in the femur: implications on pathophysiology. Singap Med J.

[36] Martelli S, Pivonka P, Ebeling PR (2014). Femoral shaft strains during daily activities: implications for atypical femoral fractures. Clin Biomech (Bristol, Avon).

[37] Koch J (1917). The laws of bone architecture. Am J Anat.

[38] Lim HS, Kim CK, Park YS, Moon YW, Lim SJ, Kim SM (2016). Factors associated with increased healing time in complete femoral fractures after long-term bisphosphonate therapy. J Bone Joint Surg Am.

